# Draft Genome Sequence of *Vibrio toranzoniae* Strain CECT 7225^T^

**DOI:** 10.1128/genomeA.00212-16

**Published:** 2016-03-31

**Authors:** Aide Lasa, Cynthia J. Gibas, Jesus L. Romalde

**Affiliations:** a Departamento de Microbiología y Parasitología, CIBUS–Facultad de Biología. Universidade de Santiago de Compostela, Santiago de Compostela, Spain; b Department of Bioinformatics and Genomics, The University of North Carolina at Charlotte, Charlotte, North Carolina, USA

## Abstract

*Vibrio toranzoniae* (CECT 7225^T^) was isolated from healthy reared carpet shell clams in Galicia (Northwest Spain). In addition, this species has been recently identified as a potential pathogen of red conger eel in Chile. The draft genome sequence has 4.5 Mbp, a G+C content of 43.9%, and >3,800 protein-coding genes.

## GENOME ANNOUNCEMENT

*Vibrio toranzoniae*, isolated from reared carpet shell clams (*Ruditapes decussatus*) in Northwest Spain, forms part of the *Splendidus* clade (1). The *Splendidus* clade has been divided into 15 species, making this group the most diverse among those of the *Vibrio* genus (2). Recently, *V. toranzoniae* was identified as the causative agent of mortality of cultured red conger eel in Chile at an experimental rearing system (3). Studies on the diversity of *Vibrio* species found that the *Splendidus* clade was the predominating group associated with bivalves. In fact, Kwan and Bolch (4) found that *V. toranzoniae* was the dominant group associated with spat tanks in a commercial mussel hatchery in Australia. It is well known that some members of the *Splendidus* clade are considered pathogenic to shellfish (oysters, clams, scallops, and mussels) (5).

Genomic DNA of *V. toranzoniae* CECT 7225^T^ was extracted using the QIAamp DNA minikit (Qiagen), according to the manufacturer’s protocol. The purified DNA was used to prepare a library, according to the Illumina TruSeq DNA sample prep protocol (Illumina, San Diego, CA). A 100-bp paired-end sequencing run of the library was performed using a HiSeq 2500 instrument (Illumina). The reads were trimmed using Trimmomatic 0.32 (6). Genome assembly was performed using SPAdes 3.6.1 (7), and QUAST (8) was used to evaluate the assembly. The subsequent assembly produced 165 contigs (>200 bp), with a G+C content of 43.93% and total genome size of 4,537,316 bp. The *N*_50_ contig size was 221,494 bp, with the largest contig being 645,319 bp.

Annotation was performed by the NCBI Prokaryotic Genome Annotation Pipeline (PGAP) (9). Additionally, the genomes were analyzed on the Rapid Annotations using Subsystems Technology (RAST) server (10). The total number of determined genes was 4,068, with a total of 3,857 coding sequences (CDSs). The genome of this strain revealed 74 pseudogenes, 94 tRNAs, 46 rRNA genes, and 1 noncoding RNA (ncRNA). According to the annotation tool employed, coding sequences for virulence factors and defense genes, such as bacteriocins and genes resistant to fluoroquinolones and tetracycline, were found. Also, capsule and extracellular polysaccharides were found, as well as siderophore genes and genes for the mechanisms of iron acquisition and metabolism. Dormancy and persistence features were also found, together with quorum sensing and biofilm formation, which would allow the bacterium to persist under unfavorable conditions. In addition, we found 145 coding sequences related to stress responses, such as osmotic and oxidative stress, cold and heat shock stress responses, and the glutathione-dependent pathway of formaldehyde detoxification.

This genome sequence will be valuable for comparative genomic studies and searches of virulence factors, thus expanding the understanding of this potential pathogen for fish.

### Nucleotide sequence accession numbers.

This whole-genome shotgun project has been deposited at DDBJ/EMBL/GenBank under the accession no. LMXU00000000. The version described in this paper is version LMXU01000000.

## References

[1] Lasa A, Diéguez AL, Romalde JL 2013 *Vibrio toranzoniae* sp. nov., a new member of the *Splendidus* clade in the genus *Vibrio*. Syst Appl Microbiol 36:96–100. doi:10.1016/j.syapm.2012.11.005. 23280322

[2] Sawabe T, Ogura Y, Matsumura Y, Feng G, Amin AR, Mino S, Nakagawa S, Sawabe T, Kumar R, Fukui Y, Satomi M, Matsushima R, Thompson FL, Gomez-Gil B, Christen R, Maruyama F, Kurokawa K, Hayashi T 2013 Updating the *Vibrio* clades defined by multilocus sequence phylogeny: proposal of eight new clades, and the description of *Vibrio tritonius* sp. nov. Front Microbiol 4:414. doi:10.3389/fmicb.2013.00414. 24409173 PMC3873509

[3] Lasa A, Avendaño-Herrera R, Estrada JM, Romalde JL 2015 Isolation and identification of *Vibrio toranzoniae* associated with diseased red conger eel (*Genypterus chilensis*) farmed in Chile. Vet Microbiol 179:327–331. doi:10.1016/j.vetmic.2015.06.003. 26072371

[4] Kwan TN, Bolch CJ 2015 Genetic diversity of culturable *Vibrio* in an Australian blue mussel *Mytilus galloprovincialis* hatchery. Dis Aquat Organ 116:37–46. doi:10.3354/dao02905. 26378406

[5] Romalde JL, Dieguez AL, Lasa A, Balboa S 2014 New *Vibrio* species associated to molluscan microbiota: a review. Front Microbiol 4:413. doi:10.3389/fmicb.2013.00413. 24427157 PMC3877837

[6] Bolger AM, Lohse M, Usadel B 2014 Trimmomatic: a flexible trimmer for Illumina sequence data. Bioinformatics 30:btu170. doi:10.1093/bioinformatics/btu170. PMC4103590 24695404

[7] Nurk S, Bankevich A, Antipov D, Gurevich AA, Korobeynikov A, Lapidus A, Prjibelsky AD, Pyshkin A, Sirotkin A, Sirotkin Y, Stepanauskas R, McLean JS, Lasken R, Clingenpeel SR, Woyke T, Tesler G, Alekseyev MA, Pevzner PA 2013 Assembling single-cell genomes and mini-metagenomes from chimeric MDA products. J Comput Biol 20:714–737. doi:10.1089/cmb.2013.0084. 24093227 PMC3791033

[8] Gurevich A, Saveliev V, Vyahhi N, Tesler G 2013 QUAST: quality assessment tool for genome assemblies. Bioinformatics 29:1072–1075. doi:10.1093/bioinformatics/btt086. 23422339 PMC3624806

[9] Tatusova T, Ciufo S, Fedorov B, O’Neill K, Tolstoy I 2014 RefSeq microbial genomes database: new representation and annotation strategy. Nucleic Acids Res 42:D553–D559. doi:10.1093/nar/gkt1274. 24316578 PMC3965038

[10] Aziz RK, Bartels D, Best AA, DeJongh M, Disz T, Edwards RA, Formsma K, Gerdes S, Glass EM, Kubal M, Meyer F, Olsen GJ, Olson R, Osterman AL, Overbeek RA, McNeil LK, Paarmann D, Paczian T, Parrello B, Pusch GD, Reich C, Stevens R, Vassieva O, Vonstein V, Wilke A, Zagnitko O 2008 The RAST server: Rapid Annotations using Subsystems Technology. BMC Genomics 9:75. doi:10.1186/1471-2164-9-75. 18261238 PMC2265698

